# Draft Genome Sequences of Clinical *Vibrio parahaemolyticus* Strains Isolated in Maryland (2010–2013)

**DOI:** 10.1128/genomeA.00776-14

**Published:** 2014-08-07

**Authors:** Julie Haendiges, Ruth Timme, Marc Allard, Robert A. Myers, Justin Payne, Eric W. Brown, Peter Evans, Narjol Gonzalez-Escalona

**Affiliations:** a Department of Health and Mental Hygiene (DHMH), Baltimore, Maryland, USA; b Center for Food Safety and Applied Nutrition, Food and Drug Administration (CFSAN-FDA), College Park, Maryland, USA

## Abstract

*Vibrio parahaemolyticus* is the leading cause of food-borne illnesses associated with the consumption of raw shellfish worldwide. Here, we report 45 draft genomes of *V. parahaemolyticus*. Thirty-five of them are strains that were isolated from clinical cases in the state of Maryland from 2010 to 2013. The remaining 10 strains were historical isolates, isolated mostly from the West Coast of the United States during the period of 1988 to 2004. The availability of these genomes will allow for future phylogenetic analyses with other *V. parahaemolyticus* strains.

## GENOME ANNOUNCEMENT

*Vibrio parahaemolyticus* is a natural inhabitant of temperate and tropical coastal waters and is the leading cause of seafood-borne gastroenteritis in the United States (1). Cases of the illness are usually associated with eating raw or undercooked seafood. Strains of *V. parahaemolyticus* carrying genes for thermostable direct hemolysin (*tdh*) and/or thermostable direct hemolysin-related hemolysin (*trh*) are associated pathogenic strains (2) and represent <1% of the environmental *V. parahaemolyticus* strains (3). During the last two decades, *V. parahaemolyticus* infections and outbreaks have increased in number throughout the world. Most of these new cases belong to the pandemic clonal complex 3 (CC3) (4–7).

The emergence of CC3 has elevated public health concerns of the worldwide spread of *V. parahaemolyticus*, previously uncharacteristic of this pathogen. The existence of other *V. parahaemolyticus* CCs (CC36 and CC34) has been observed among the coastal United States strains (4). Although infections in the United States are typically caused by strains from the CC36 endemic to the West Coast (4, 8), an outbreak in Maryland in August 2012 (7) was caused by strains belonging to the pandemic clonal complex 3 (CC3). A total of 56 cases associated with *V. parahaemolyticus* were reported between 2010 and 2013 in the state of Maryland. We sequenced 35 of these *V. parahaemolyticus* outbreak strains isolated from those cases to better understand the potential changes in the *V. parahaemolyticus* populations on this coastal state (Table 1). Ten additional historical *V. parahaemolyticus* strains from different sources (clinical and environmental) were also sequenced (Table 1).

**TABLE 1:** List of the *V. parahaemolyticus* strains sequenced in this study and their GenBank accession no.

Strains	WGS accession no.^*a*^	CFSAN no.	No. of contigs	ST	State of isolation	Yr of isolation	Source^*b*^
VP1	JNSM00000000	CFSAN007429	285	631	MD	2012	C
VP8	JNSN00000000	CFSAN007430	298	631	MD	2012	C
VP9	JNSO00000000	CFSAN007431	278	631	MD	2012	C
VP31	JNSP00000000	CFSAN007432	273	631	MD	2013	C
VP35	JNSQ00000000	CFSAN007433	319	631	MD	2013	C
VP41	JNSR00000000	CFSAN007434	233	631	MD	2013	C
VP44	JNSS00000000	CFSAN007435	223	631	MD	2013	C
VP45	JNST00000000	CFSAN007436	214	631	MD	2013	C
VP2	JNSU00000000	CFSAN007437	243	651	MD	2012	C
VP3	JNSV00000000	CFSAN007438	212	652	MD	2012	C
VP4	JNSW00000000	CFSAN007439	184	653	MD	2012	C
VP34	JNSX00000000	CFSAN007440	249	653	MD	2013	C
VP5	JNSY00000000	CFSAN007441	286	113	MD	2012	C
VP7	JNSZ00000000	CFSAN007442	287	113	MD	2012	C
VP11	JNTA00000000	CFSAN007443	283	113	MD	2012	C
VP6	JNTB00000000	CFSAN007444	135	677	MD	2012	C
VP10	JNTC00000000	CFSAN007445	203	43	MD	2012	C
VP13	JNTD00000000	CFSAN007446	137	678	MD	2012	C
VP14	JNTE00000000	CFSAN007447	214	162	MD	2012	C
VP15	JNTF00000000	CFSAN007448	232	679	MD	2012	C
VP16	JNTG00000000	CFSAN007449	147	3	MD	2012	C
VP17	JNTH00000000	CFSAN007450	157	3	MD	2012	C
VP18	JNTI00000000	CFSAN007451	129	3	MD	2012	C
VP19	JNTJ00000000	CFSAN007452	304	8	MD	2010	C
VP20	JNTK00000000	CFSAN007453	186	8	MD	2010	C
VP39	JNTL00000000	CFSAN007455	217	896	MD	2013	C
VP12	JNTM00000000	CFSAN006129	284	36	MD	2012	C
VP32	JNTN00000000	CFSAN006131	281	36	MD	2013	C
VP33	JNTO00000000	CFSAN006132	283	36	MD	2013	C
VP36	JNTP00000000	CFSAN006133	276	36	MD	2013	C
VP38	JNTQ00000000	CFSAN006134	215	36	MD	2013	C
VP40	JNTR00000000	CFSAN006135	250	36	MD	2013	C
VP42	JNTS00000000	CFSAN007460	279	36	MD	2013	C
VP43	JNTT00000000	CFSAN007461	185	36	MD	2013	C
VP30	JNTV00000000	CFSAN006130	269	36	MD	2013	C
029-1(b)	JNTW00000000	CFSAN001611	120	36	OR	1997	E
48057	JNTX00000000	CFSAN001612	111	36	WA	1990	C
K1198	JNTY00000000	CFSAN001614	130	59	AK	2004	E
10292	JNTZ00000000	CFSAN001617	126	50	WA	1997	C
48291	JNUA00000000	CFSAN001618	105	36	WA	1990	C
F11-3A	JNUB00000000	CFSAN001619	104	36	WA	1988	E
NY-3483	JNUC00000000	CFSAN001620	125	36	NY	1998	E
K1203	JNUD00000000	CFSAN001173	209	59	AK	2004	E
98-513-F52	JNUE00000000	CFSAN001160	120	34	LA	1998	E
10290	JNUF00000000	CFSAN001613	151	36	WA	1997	C

a WGS- NCBI whole-genome shotgun assembly database.

b C, clinical; E, environmental; ST, sequence type.

The genomes were sequenced using Ion Torrent, and *in silico* multilocus sequence typing (MLST) (4) showed that these isolates exhibited diverse sequence types (STs) (Table 1). DNA from each strain was isolated from overnight cultures with the DNeasy blood and tissue kit (Qiagen, Valencia, CA). The genomes were sequenced using the Ion Torrent (PGM) sequencing system. The 36 strains from Maryland were sequenced using 300-bp read chemistry (Life Technologies), while the 10 historical strains were sequenced using 200-bp read chemistry, according to manufacturer’s instructions, at 17 to 68× coverage using the Ion PGM 200 or 300 sequencing kit, respectively, depending of the template used, according to manufacturer’s instructions. The genomic sequence contigs for each strain were *de novo* assembled using the CLC Genomics Workbench version 5.5.1 (CLC bio, Germantown, MD, USA). The G+C mol% of the strains was between 45.1 and 45.4%, which is similar to the reported G+C contents for other *V. parahaemolyticus* strains. The sequences were annotated using the NCBI Prokaryotic Genomes Automatic Annotation Pipeline (http://www.ncbi.nlm.nih.gov/genomes/static/Pipeline.html) (9). A detailed report of a full comparative analysis between these *V. parahaemolyticus* genomes will be included in a future publication.

This large data release contributes to the efforts of a newly created *V. parahaemolyticus* BioProject (no. PRJNA245882) at the NCBI, spearheaded by the Center for Food Safety and Applied Nutrition (CFSAN)-FDA and the Department of Health and Mental Hygiene (DHMH) of the state of Maryland, in order to improve the detection of new strains or track the emergence of new clonal strains in geographical regions where these strains are not endemic.

### Nucleotide sequence accession numbers.

The draft genome sequences of the 45 *V. parahaemolyticus* strains are available in GenBank under the accession numbers listed in Table 1.
